# Nanozyme-natural enzymes cascade catalyze cholesterol consumption and reverse cancer multidrug resistance

**DOI:** 10.1186/s12951-022-01406-9

**Published:** 2022-05-02

**Authors:** Bin Du, Mei Zheng, Huizhen Ma, Jingshu Huang, Qingqing Jiao, Yimeng Bai, Mengmeng Zhao, Jie Zhou

**Affiliations:** 1 School of Pharmaceutical Sciences, Zhengzhou University, 100 Science Road, Zhengzhou, 450001 People’s Republic of China; 2 Key Laboratory of Targeting Therapy and Diagnosis for Critical Diseases, 100 Science Road, Zhengzhou, 450001 Henan Province People’s Republic of China

**Keywords:** Cholesterol oxidase, Fluidity, Cholesterol, Lipid raft, Chondroitin sulfate, Apoptosis, Reverse drug resistance

## Abstract

Multidrug resistance is still a major obstacle to cancer treatment. The most studies are to inhibit the activity of the drug transporter P-glycoprotein (P-gp), but the effect is not ideal. Herein, a nanosystem was built based on cascade catalytic consumption of cholesterol. Cholesterol oxidase (natural enzyme, COD) was immobilized on the carrier (NH_2_-MIL-88B, MOF) through amide reaction, COD catalyzed the consumption of cholesterol, the reaction product H_2_O_2_ was further produced by the MOF with its peroxidase-like activity to produce hydroxyl radicals (•OH) with killing effect. Due to the high expression of CD44 receptor on the surface of tumor cells, we encapsulated chondroitin sulfate gel shell (CS-shell) with CD44 targeting and apoptosis promoting effect on the surface of DOX@MOF-COD nanoparticles, which can accurately and efficiently deliver the drugs to the tumor site and improve the effect of reversing drug resistance. Taking drug-resistant cell membrane as "breakthrough", this paper will provide a new idea for reversing multidrug resistance of tumor.

**Graphical Abstract:**

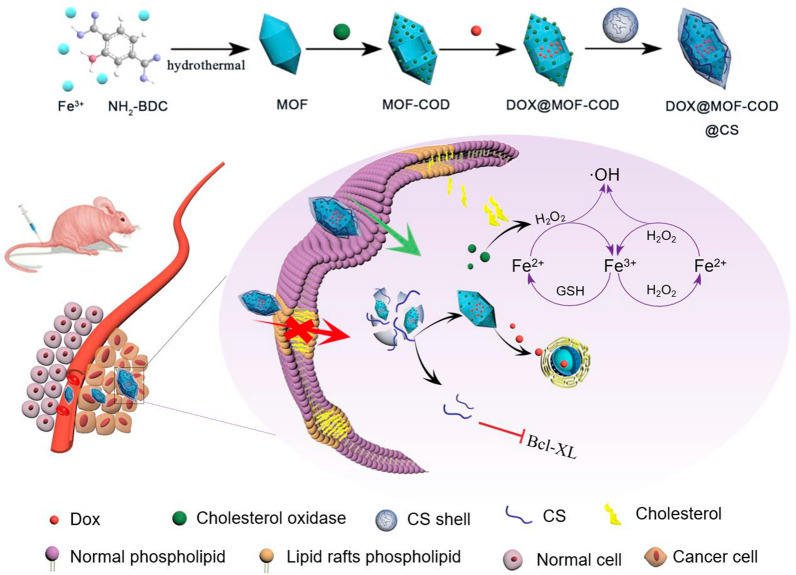

**Supplementary Information:**

The online version contains supplementary material available at 10.1186/s12951-022-01406-9.

## Introduction

Chemotherapy failure is commonly attributed to multidrug resistance (MDR) [1–4]. At present, the most extensive researches are mainly to inhibit the expression of the drug transporter P-glycoprotein (P-gp) [5–7]. The ATP binding cassette (ABC) superfamily is one of the most involved membrane protein families, including P-gp (ABCB1) [8], MRP1 (ABCC1) [9], MRP2 (ABCC2) etc., [10, 11]. Because P-gp has a very wide range of capture properties, most drugs are P-gp substrates, such as P-gp can transport doxorubicin, paclitaxel, cisplatin, colchicine, etc., [12]. Therefore, for a long time the research is aimed at the activity of the drug efflux pump P-gp. Three generations of molecular inhibitors have been lately developed for the efflux pump P-gp [13, 14]. However, most P-gp inhibitors are not effective, this is mainly because P-gp is easier to locate in cholesterol rich drug-resistant cell membrane, and it only reverses drug resistance in one way [15, 16]. Therefore, overcoming drug resistance from the physiological barrier is an urgent problem to be solved.

Penetrating the cell membrane is a prerequisite for drug delivery. As a barrier between the cell and the extracellular microenvironment, the cell membrane contains hydrophobic lipids (phosphatidylcholine, phosphatidylserine, cholesterol, sphingolipids, etc.,) and proteins [17]. Among them, cholesterol is a sterol with four hydrocarbon rings that regulate membrane fluidity and lipid raft formation. Thus, cholesterol-rich membranes will increase mechanical stiffness and reduce membrane fluidity [18–20]. Cell membrane can respond differently to external stimuli (such as those from chemotherapeutic drugs), which depends on changes in the composition of membrane lipids [6]. Cholesterol content in drug-resistant cell membranes is usually increased [21, 22]. Therefore, cholesterol-rich membranes will increase mechanical stiffness and reduce membrane fluidity. From a diffusion perspective, the decrease of membrane fluidity significantly affects the ability of drugs to penetrate the biological barrier into the cell. In addition, lipid raft domains are defined as cholesterol-rich and spingolipid-rich regions of the cell membrane that are generally more ordered [23]. Therefore, changes in cholesterol content have a great impact on the fluidity of cell membranes. How to construct a nano-delivery system to consume cholesterol to regulate fluidity is a key issue that needs to be resolved urgently to reverse tumor multidrug resistance.

Cholesterol oxidase is the most important catalytic enzyme for cholesterol metabolism [24, 25]. Huang et al. used cholesterol oxidase to prepare a biosensor to monitor cholesterol concentration [26, 27]. Based on this, this article uses cholesterol oxidase to consume cellular cholesterol and catalyze cholesterol to produce cholestenone and hydrogen peroxide. Through the catalytic oxidation of cholesterol, cholesterol can be effectively reduced, so the fluidity of cell membrane can be enhanced.

Since cholesterol oxidase activity is easily destroyed, the selection of the carrier is extremely critical. The metal–organic framework (MOF) consists of inorganic units and organic ligands, also known as porous coordination polymers [28–30]. This material has the characteristics of high porosity, large specific surface area, high loading capacity and strong molecular catalytic center [31–33]. In addition, MOF has superior peroxidase activity and can produce hydroxyl free radicals to kill tumor cells. Chen et al. used nanoenzymes with peroxidase activity to catalyze low H_2_O_2_ to produce hydroxyl radicals to kill bacteria [34]. By confining passivated gold nanoparticles and multiple cerium (IV) complexes on the surface of the colloidal magnetic Fe_3_O_4_/SiO_2_ core/shell particles, an artificial enzyme with DNase-like activity was obtained, showing better operational stability and recyclability, and significantly enhanced the efficiency of traditional antibiotics to kill bacteria [35]. In the paper, cholesterol oxidase was loaded on the metal organic framework NH_2_-MIL-88B, and the abundant amino groups of MOF were used to successfully immobilize cholesterol oxidase on MOF through amide reaction, which improved the tolerance of the enzyme to pH and temperature [36].

It is very important to construct an efficient and safe drug delivery system. Chondroitin sulfate (CS) is an endogenous molecule with good safety [37–40]. CS has a strong affinity for the CD44 receptor (high expression of CD44 in the cancer cells), it can accurately target the tumor site [41, 42]. More importantly, as an anti-inflammatory drug, CS inhibits the synthesis of the pro-inflammatory enzyme COX-2, reduces the level of the anti-apoptotic protein Bcl-XL, and further increases the sensitivity of tumor cells to chemotherapeutic drugs [43, 44].

**Scheme 1:**
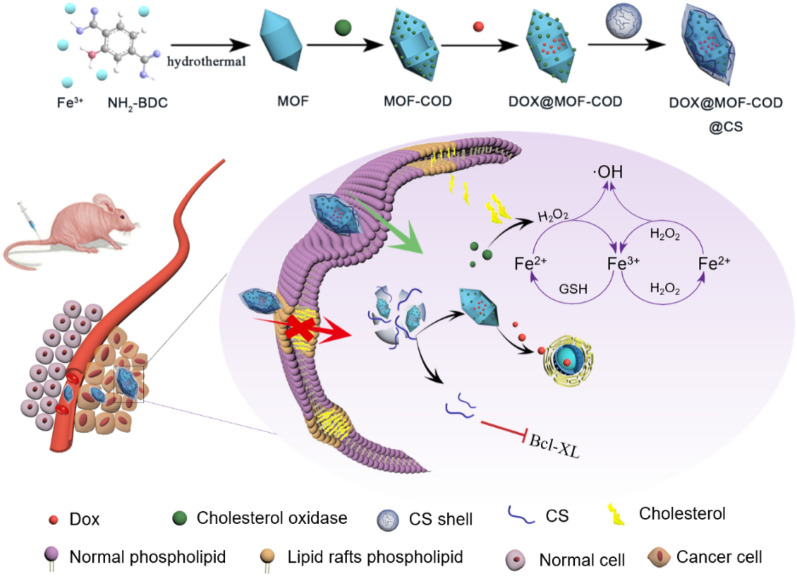
Preparation of nanocarriers based on cascade catalysis and tumor region specific targeting for efficient delivery and reversal of multidrug resistance

Doxorubicin (DOX) can easily lead to chemotherapy resistance. Therefore, how to achieve efficient delivery of chemotherapeutic drugs is the key to reversing drug resistance. Li et al. used fluorinated polymers to encapsulate the chemotherapeutic drugs cisplatin or paclitaxel to achieve a therapeutic effect [45, 46]. In this paper, DOX is used as a model drug, loaded on MOF-COD nanoparticles and an enzymatic reaction-based DOX@MOF-COD@CS nanosystem (Scheme 1) is cleverly constructed. Among them, CS shell contains disulfide bonds, which can play a role in response to the cleavage of glutathione (GSH) and release drug-loaded nanoparticles to initiate cascading catalytic reactions and apoptosis processes, respectively, thus effectively improving therapeutic efficacy. The main mechanisms are: (1) In response to the increase in cholesterol content of drug-resistant cell membranes and the decrease in fluidity, cholesterol oxidase is used to reduce cholesterol, thereby enhancing cellular uptake; (2) Cholesterol oxidase catalyzes cholesterol metabolite H_2_O_2_, which can be further catalyzed by nanozyme MOF to produce •OH. The conversion of cholesterol to •OH is beneficial to solve the drug resistance, which has cancer cell killing effect, and turn “waste” into “treasure” to maximize drug resistance; and (3) CS shell inhibits the synthesis of the pro-inflammatory enzyme COX, leading to the down-regulation of the anti-apoptotic protein Bcl-XL, thereby increasing the sensitivity of tumor cells to chemotherapeutic drugs.

## Materials and methods

### Materials

FeCl_3_·6H_2_O and CH_3_COOH (Analytically pure) were purchased from Tianjin Zhiyuan Chemical Reagent Co., Ltd. (Tianjin, China). Pluronic F127, EDCI and NHS (98% purity) were from Shanghai Aladdin Reagent Co., Ltd. (Shanghai, China). Cholesterol oxidase was purchased from Shanghai Macleans Biochemical Technology Co., Ltd. (Shanghai, China). Adriamycin hydrochloride (purity > 98%) was bought from Dalian Meilun Biological Co., Ltd. (Dalian, China). Chondroitin sulfate (purity > 98%) and cystamine dihydrochloride (purity > 98%) were from Shanghai Yuanye Biotechnology Co., Ltd. (Shanghai, China).

### Cells and animals

Human breast cancer cells MCF-7/ADR were purchased from Jiangsu KGI Biotechnology Co., Ltd. (Jiangsu, China). BALB/c female nude mice (16–20 g, 5 weeks, SPF grade) were provided by Beijing Sibeifu Biotechnology Co., Ltd. (Beijing, China).

### Synthesis of DOX@MOF-COD@CS nanoparticles

0.32 g Pluronic F127 was dissolved in 26.68 mL ultrapure water and 0.357 g FeCl_3_·6H_2_O was dissolved in 3.32 mL water. The two solutions were mixed and stirred for 1.5 h, and then 0.6 mL of acetic acid was added. After 1.5 h, 120 mg H_2_N-BDC was added. After the reaction mixture was stirred at room temperature for 4 h, it was transferred to an autoclave (110 °C) for crystallization for 24 h. The dark brown solid product was recovered and washed with ethanol several times (at least six times) to remove the surfactant and excess reactants. The obtained solid was put into a vacuum drying oven at 50 °C for 1 day to obtain NH_2_-MIL-88B powder.

Cholesterol oxidase (10.0 mg) was dissolved in 10.0 mL MES buffer solution (pH 5), and 120.0 mg 1-(3-dimethylaminopropyl) -3-ethylcarbodiimine hydrochloride (EDCI) and 150.0 mg N-hydroxy-succinimide (NHS) were successively added into the enzyme solution. After being activated at 37 °C for 15 min in a shaker with 100 rpm, MOF powder (30.0 mg) was added into 10 mL phosphate solution and ultrasound was carried out for 15 min to make it evenly dispersed. The two solutions were mixed and reacted overnight. After that, MOF-COD nanoparticles were prepared by 8000*g* centrifugation for 5 min. Doxorubicin hydrochloride (5.0 mg) was dissolved in 5 mL phosphate buffer. MOF-COD was evenly dispersed in 5 mL phosphate buffer solution. The two were mixed and stirred in a magnetic mixer for 24 h. Then, the solution was centrifuged at 8000*g* for 5 min to remove the free DOX to obtain DOX@COD-MOF nanoparticles.

Chondroitin sulfate (40.0 mg) was fully dissolved in 10 mL MES buffer (pH 5), EDCI and NHS were activated for 15 min, and 10.0 mg cysteamine dihydrochloride was dissolved in 10 mL phosphate buffer. The two solutions were mixed evenly and reacted overnight. After 24 h of dialysis in the dark, the white product was vacuum dried. DOX@MOF-COD nanoparticles were dispersed in a phosphate buffer solution, and the CS solution was added dropwise to the above solution under dark conditions, and stirred in a magnetic stirrer for 8 h. Then the DOX@MOF-COD@CS nanosystem was obtained by ultrasound.

### In vitro enzyme-like properties

3, 3′, 5, 5′-tetramethylbenzidine (TMB), o-phenylenediamine (OPD), 2, 2′-azido-bis (3-ethylbenzothiazoline-6-sulfonic acid (ABTS) was used as an indicator. The experiment was divided into three groups: MOF + H_2_O_2_ + indicator, MOF + indicator, H_2_O_2_ + indicator. 2.94 mL NaAc-HAC buffer (pH 4) was preheated at 37 °C for 10 min, then TMB was dissolved in anhydrous ethanol. 20 μL of TMB, OPD, ABTS (1 mM), H_2_O_2_ (200 μM) and MOF (40 μg mL^−1^) solutions were added sequentially. After reacting for 30 min, the three sets of solutions were scanned at full wavelength using an ultraviolet–visible spectrophotometer.

Terephthalic acid (PTA) was used as an indicator, 2.94 mL NaAc-HAC buffer (pH 4) was preheated at 37 °C for 10 min, then 20 μL PTA solution (1 mM), H_2_O_2_ (200 μM) and MOF (40 μg mL^−1^) were added successively, and the reaction time was 30 min. All three experiment groups were scanned at full wavelength with a fluorescence spectrophotometer.

### Cell resistance determination

MCF-7 and MCF-7/ADR cells were seeded into 96 well plates and DOX solutions with different concentration gradients were added. The concentration gradients of DOX were 0.2 to 25.6 μg mL^−1^ in MCF-7 cells, and 1.25–100 μg mL^−1^ in MCF-7/ADR cells, respectively. The cells were incubated for 24 and 48 h, the cell survival rate was detected by SRB assay. Resistance index (RI) value could be calculated by IC50 of resistant cells/IC50 of sensitive cells.

### Cell uptake

MCF-7/ADR cells were seeded in 2 × 10^5^ cells/well in a six-well plate, and incubated for 24 h. After adding 2 mL of DOX, DOX@MOF-COD and DOX@MOF-COD@CS nanoparticles, respectively (concentration of DOX: 10 μg mL^−1^), incubation was conducted (1, 2, 4, 8 h). The culture medium was discarded, the cells in PBS were washed for three times, 1 mL of 4% paraformaldehyde was added and placed in the incubator for 20 min. After washing the cells for three times with PBS, 1 mL DAPI solution (5 μg mL^−1^) was added and stained for 15 min. After washing the cells with PBS for 3–4 times, the cells were observed under confocal laser scanning microscope (CLSM).

### Investigation of cholesterol content

MCF-7/ADR cells were seeded in a six-well plate at 2 × 10^6^ cells/well and incubated overnight. MOF-COD, DOX@MOF-COD@CS (concentration of DOX: 10 μg mL^−1^) were added and diluted with culture medium. M-β-CD was the positive control group (5 mM). After incubation for 24 h, the medicated media were discarded, the cells were washed 3 times with PBS, and the cholesterol concentration of each group was determined by the cholesterol kit.

### Fluidity measurement of cell membrane

The concentration of MCF-7/ADR cell suspension was adjusted to 2 × 10^5^/well and inoculated in the six-well plate. The cells were cultured overnight in an incubator, and M-β-CD (positive control, 5 mM), MOF-COD and DOX@MOF-COD@CS nanoparticles (DOX concentration was 10 μg mL^−1^) were diluted in medium. After incubation for 2 h, the medium was discarded, the cells were washed with PBS for three times, and 1 mL trypsin was added to make cell suspension. The cell suspension was incubated with 1-pyridinedienoic acid (final concentration: 2 μM) for 5 min in darkness. In the fluorescence spectrophotometer, the excitation wavelength of 380 nm and the emission wavelength of 380–580 nm were used to scan the fluorescence spectra, and the fluidity of each group was compared according to the ratio of excimer 475 nm to pyrene monomer 397 nm.

### Membrane lipid raft structure determination

MCF-7/ADR cells were seeded into 6-well plates with 2 × 10^5^ cells per well and cultured for 24 h. The medium was discarded, and M-β-CD (5 mM), MOF-COD and DOX@MOF-COD@CS nanoparticles (DOX concentration was 10 μg mL^−1^) were diluted with the medium, the control group was set, and incubated for 24 h. The culture medium was discarded, the cells were washed with PBS for three times, and 1 mL AF488-CTB (5 μg mL^−1^) was added to each well. The cells were placed on ice and incubated in the dark. The cells were washed three times with PBS and fixed with 1 mL 4% paraformaldehyde for 15 min. After the cells were washed three times with PBS, 1 mL DAPI was added for staining (15 min, 37 °C), washed 3–5 times with PBS, observed and photographed under a confocal laser microscope.

### Western blotting analysis

MCF-7/ADR cells were treated with DOX@MOF-COD, DOX@MOF-COD@CS nanoparticles for 24 h, washed with PBS, added lysate to lyse cells on ice and extracted cell protein. The contents of Bcl-XL, COX-2 and P-gp were quantitatively determined by western blot.

### Biodistribution of DOX@MOF-COD@CS nanoparticles in nude mice

MCF-7/ADR cells were inoculated into the axilla of the right forelimb of 5-week-old nude mice in a quantity of 1 × 10^7^ cells/100 μL, and the tumor growth and status were regularly observed. If the tumor volume reaches 60 mm^3^, a nude mouse model of tumor-bearing mice was successfully established. IR783 was encapsulated into the carrier as a fluorescent dye to prepare IR783@MOF-COD@CS nanoparticles. Two groups of nude mice were injected through the tail vein, and the distributions of IR783@MOF-COD@CS nanoparticles and free IR783 in the body were observed at different time points (1, 2, 4, 8, 12 and 24 h) using a near-infrared imager. The anesthetic pentobarbital sodium solution (7 mg kg^−1^) was intraperitoneally injected and fixed on a live imaging device to take pictures and record the fluorescence distribution.

In addition, the fluorescence distributions of the isolated tissues and tumors were investigated. Nude mice were sacrificed after 12 h of injection, and the organs (spleen, liver, lung, kidney, heart) and tumors were collected. The fluorescence distributions in tissues and organs in *vitro* were photographed by in an imaging system.

### Tumor inhibition study

When the tumor volume reached 100 mm^3^, the nude mice were randomly divided into 7 groups with 6 mice in each group. The groups were as follows: (1) Saline, (2) free DOX, (3) MOF, (4) MOF-COD, (5) DOX@MOF, (6) DOX@MOF-COD, and (7) DOX@MOF-COD@CS (DOX dose: 5 mg kg^−1^). The preparation of each group was injected via tail vein every other day, and the treatment ended 14 days later.

During the treatment, the normal feeding and growth of nude mice should be ensured. The nude mice were weighed and recorded before each administration. The tumor volume was calculated according to the formula: volume = (width)^2^ × length/2. After treatment, nude mice of each group were dissected, the heart, liver, spleen, lung, and kidney were soaked in 10% formalin and stained with hematoxylin and eosin. The tumor weight of nude mice was taken and the tumor inhibition rate was calculated according to the following formula: tumor inhibition rate (%) = (*V*_0_ − *V*)/*V*_0_, where *V* is the average tumor volume of different preparation groups. *V*_0_ represents the average tumor volume in the saline group.

### In vitro safety

After the treatment, blood was collected from the orbit of nude mice and placed in heparin sodium anticoagulant tube to detect and quantify the indexes of hemoglobin and creatinine. The heart, liver, spleen, lung and kidney of nude mice in each group were washed with normal saline, dried with filter paper, the weight of organs was weighed with electronic balance, and the organ index was calculated according to the following formula: organ index = organ weight (g)/nude mouse weight (g).

### Statistical analysis

All experimental data are analyzed by GraphPad Prism 8. Statistical analysis adopts *t* test and One-way ANONA analysis. *P < 0.05, **P < 0.01, ***P < 0.001.

## Results and discussion

### Preparation and characterization of DOX@MOF-COD@CS nanoparticles

**Fig. 1:**
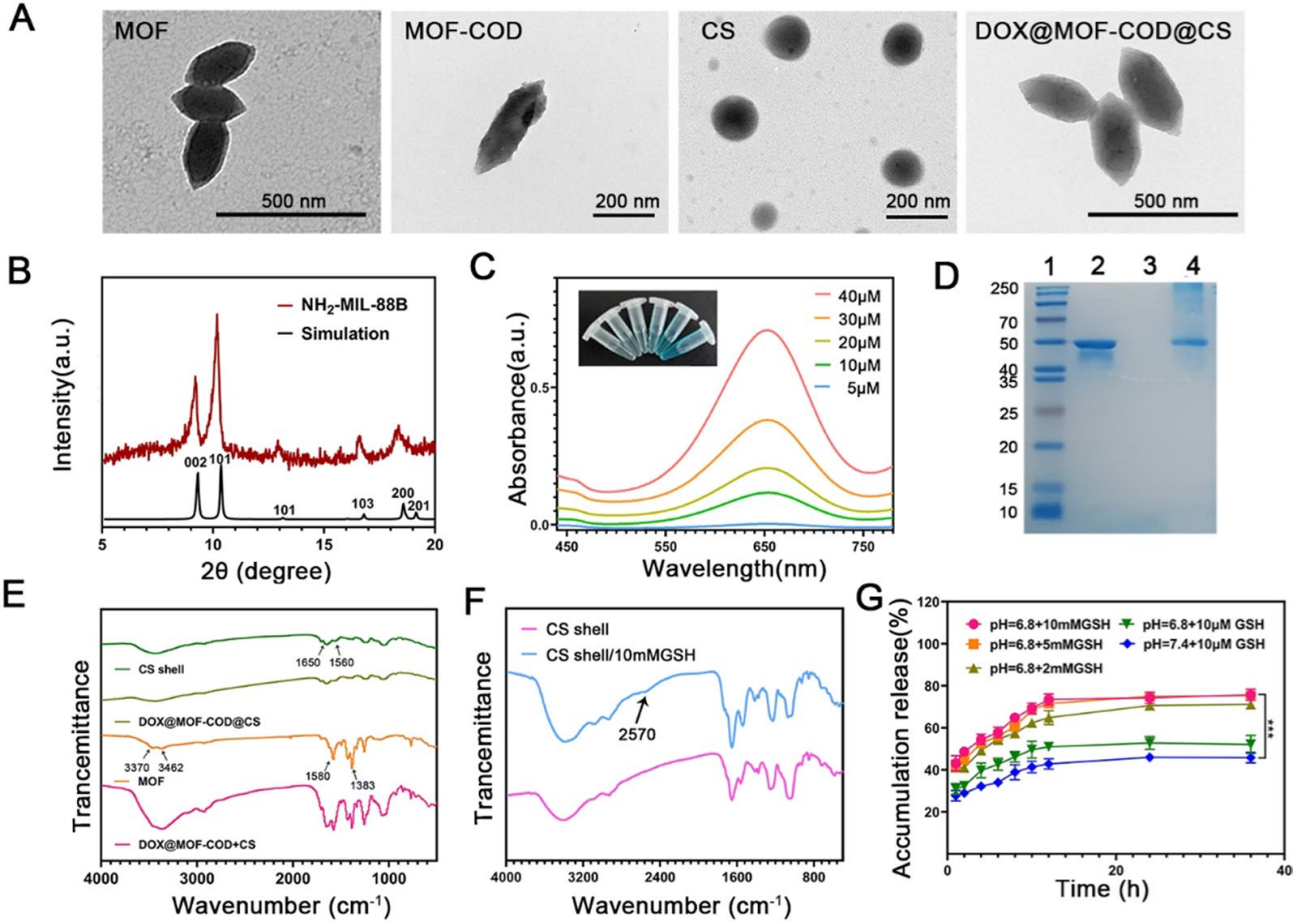
Characterization of DOX@MOF-COD@CS nanosystem. **A** TEM micrographs of the MOF, MOF-COD, CS and DOX@MOF-COD@CS nanoparticles. **B** XRD spectra of NH_2_-MIL-88B. **C** Peroxidase-like properties of MOF-COD. **D** SDS-PAGE images. 1: Marker, 2: COD (~ 50KDa), 3: MOF, 4: MOF-COD (~ 50KDa) without free COD. **E** FT-IR spectra of CS shell, DOX@MOF-COD@CS, MOF and DOX@MOF-COD + CS. **F** Infrared spectra of CS response to GSH cracking. **G** In vitro release curves of DOX. Data are presented as mean ± SD, ***P < 0.001

The MOF was synthesized by solvothermal method [46, 47]. Using acetic acid and Pluronic F127 as particle size regulators, a uniform spindle structure with a size of about 200 nm was obtained. As shown in Fig. 1A, MOF-COD has an obvious protein modification outside the MOF, and its shape was still a spindle shape, indicating that the modification of COD does not change the shape and size of the MOF. It was observed by transmission electron microscope that CS gel nanoparticles were spherical with a particle size of about 180 nm. DOX@MOF-COD@CS nanoparticles were still spindle-shaped, but these particles were rounder with a particle size of about 250 nm. The results were consistent with those measured by the nano-laser particle size analyzer (Additional file 1: Fig. S1A). The average particle sizes of MOF, MOF-COD, DOX@MOF-COD, and DOX@MOF-COD@CS nanoparticles were 215.3 ± 11.2, 226.0 ± 8.2, 244.3 ± 6.6, 266.7 ± 8.6 nm, respectively. These results were almost consistent with those of transmission electron microscope. The average potentials of MOF, MOF-COD, DOX@MOF-COD, and DOX@MOF-COD@CS nanoparticles were − 20.1 ± 0.8, − 21.3 ± 0.9, − 35.4 ± 0.5, − 30.7 ± 0.6 mV, respectively. As shown in Fig. 1A, MOF had a good crystal form, and its structure was coincident with the XRD data of MIL-88 (Cr). [48] The characteristic peaks at 2°, 10.5°, 13.2°, 17.2°, 18.2° and 20.5° indicated the successful formation of MOF crystals. The peroxidase activity of MOF was verified by color reaction (Additional file 1: Fig. S1B–E). In the presence of different concentrations of cholesterol, MOF-COD can catalyze the formation of H_2_O_2_, which was then catalyzed by MOF to generate active oxygen, and finally TMB was catalyzed. There was a maximum absorption peak at the wavelength of 650 nm, and the solution became blue (Fig. 1C). As shown in Fig. 1D, 2 represented for COD, 3 represented for MOF, 4 represented for MOF-COD (free COD had been removed). The molecular weight of COD was ~ 50KDa, MOF showed no band, and the molecular weight of MOF-COD band was ~ 50KDa, which can preliminarily prove the successful preparation of MOF-COD.

As shown in Fig. 1E, the CS shell corresponds to the amide I bond at 1650 cm^−1^, which was the stretching vibration of C=O, and the amide II bond at 1560 cm^−1^ was caused by the stretching vibration of C−N and the deformation vibration of N−H. It showed that the CS shell was formed by cross-linking of amide bonds. MOF corresponded to the stretching vibration peak of hydroxyl at 1580 cm^−1^ and 1383 cm^−1^, 3462 cm^−1^ and 3370 cm^−1^ were caused by the symmetric and asymmetric stretching absorption of primary amine groups, there was no characteristic band of protonated carboxyl group at 1700 cm^−1^, indicating that the carboxyl group has coordinated with Fe^3+^ [47, 49]. In the DOX@MOF-COD@CS group, the infrared characteristic peaks of MOF were masked, indicating that DOX@MOF-COD nanoparticles were wrapped by the CS shell. The successful loading of DOX was proved by the fluorescence emission spectra of DOX, MOF, MOF-COD and DOX@MOF-COD@CS solutions (Additional file 1: Fig. S2). In addition, the DOX@MOF-COD@CS delivery system showed good stability and security (Additional file 1: Fig. S3).

Since the concentration of GSH in the tumor environment (10 mM) is much higher than that in the normal environment (10 μM), when DOX@MOF-COD@CS nanoparticles reached tumor cells, the disulfide bonds responded to the high concentrations of GSH, CS shell cracking (Fig. 1F) [43], DOX can be slowly released from MOF. To further verify the GSH responsiveness of the preparation, this experiment simulated the pH values and GSH concentrations of tumor or normal cells in vivo, as shown in Fig. 1G, When the GSH concentration was 10 mM and pH6.8 (tumor microenvironment). The release of DOX from DOX@MOF-COD@CS nanoparticles was about 69.3% within 10 h. Under the conditions of 2 mM GSH and pH 6.8, the DOX release was about 62.4%.

### Study on reversing drug resistance in vitro by nanoparticles

**Fig. 2:**
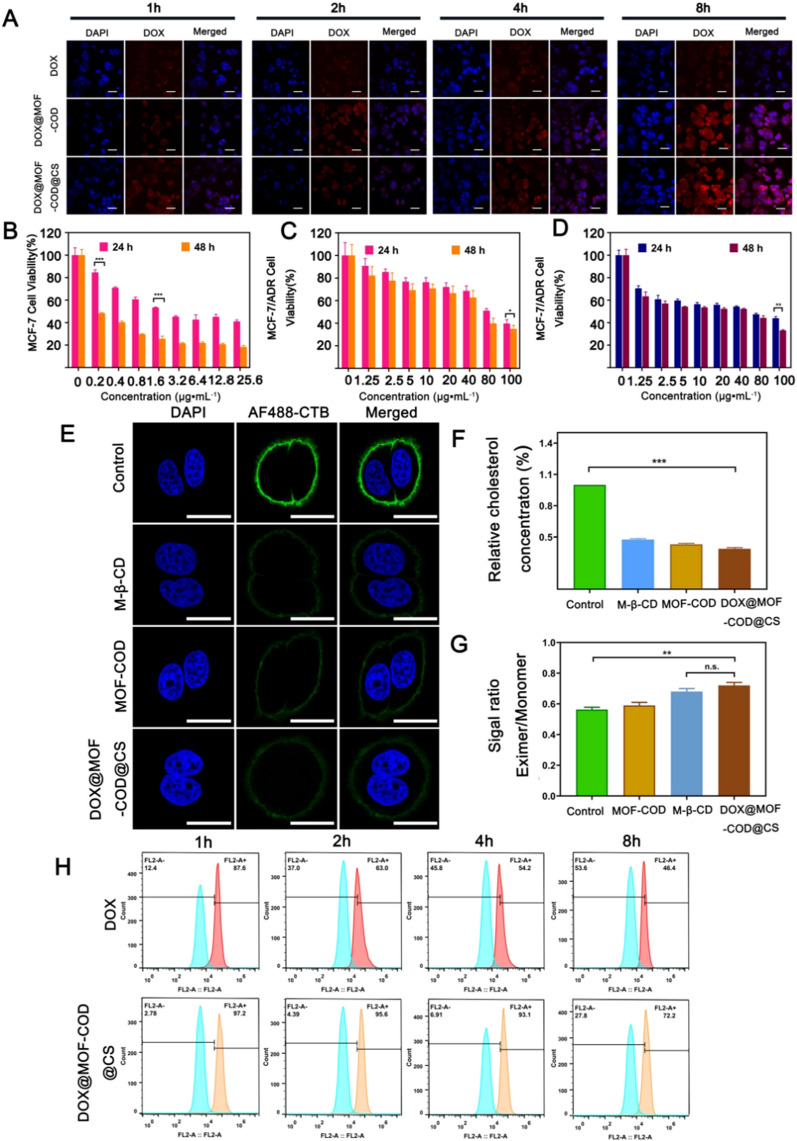
**A** DOX, DOX@MOF-COD, DOX@MOF-COD@CS nanoparticles co-incubated with MCF-7/ADR cells, the CLSM images of the drugs in the cells, scale bar: 50 μm. **B** and **C** Cell viabilities of MCF-7 and MCF-7/ADR cells incubated with DOX for 24 and 48 h (*n* = 6). **D** Cell survival rate of DOX@MOF-COD@CS nanoparticles incubated with MCF-7/ADR cells for 24 h and 48 h, respectively (*n* = 6). **E** CLSM observed membrane lipid rafts labeled with AF488-CTB, nucleus: DAPI blue fluorescent lipid rafts: green fluorescence labeled with AF-488-CTB, scale: 20 μm. **F** Determination of cholesterol content in MCF-7/ADR cells treated with Control, MOF-COD, M-β-CD and DOX@MOF-COD@CS groups. **G** Quantitative analysis of cell membrane fluidity (*n* = 3). **H** Flow cytometry analysis of DOX accumulation in MCF-7/ADR cells (*n* = 3). FL2-A: the fluorescence channel of DOX; FL2-A +: DOX fluorescence intensity increases; FL2-A−: DOX fluorescence intensity decreases. Data are presented as mean ± SD, *P < 0.05, **P < 0.01, ***P < 0.001

The uptake and excretion of DOX@MOF-COD@CS were observed in drug-resistant cells over time through CLSM. As shown in Fig. 2A, when free DOX interacted with cells for 1 h and 2 h, only weak red fluorescence was found in the cells. At 4 h, some DOX was taken up by the cells, and small amount entered the nucleus. At 8 h, the red fluorescence intensity in the cells did not increase significantly. This may be due to the fact that the DOX that entered the cells were discharged from the cells by P-gp, and the accumulation of intracellular drugs was reduced. DOX@MOF-COD nanoparticles were incubated with the cells for 4 h. Compared with free DOX, the cells showed stronger red fluorescence, which was obviously co-localized with the nuclei. At 8 h, the fluorescence intensity of intracellular DOX did not decrease significantly. Free CS was used as a blocker, the uptake of DOX@MOF-COD@CS + CS group was significantly lower than that of DOX@MOF-COD@CS group, which may be due to the fact that CS binded to the CD44 receptor on the cell surface and competitively blocked the receptor-mediated endocytosis pathway, leading to a decrease in the uptake of DOX@MOF-COD@CS nanoparticles. In order to further prove the CD44 targeting effect of DOX@MOF-COD@CS nanoparticles, these nanoparticles were incubated with hyaluronic acid, and the fluorescence intensity of DOX@MOF-COD@CS group was obviously decreased, indicating that DOX@MOF-COD@CS nanoparticles had the CD44 targeting effect (Fig. S4). Since CS shell can target the CD44 receptor on the tumor surface, after 8 h, compared with DOX@MOF-COD group, the uptake of the final preparation was further increased.

The low concentration of DOX (120 ng ml^−1^) was added into MCF-7/ADR cells for the continuous stimulation during the cell growth to maintain the cell resistance to DOX. In order to investigate the drug resistance of MCF-7/ADR cells. A series of DOX solutions were added to MCF-7 and MCF-7/ADR cells, respectively, and the sensitivities of the two kinds of cells to DOX were investigated. As shown in Fig. 2B and C, DOX had high cytotoxicity to MCF-7 cells, with an IC_50_ value of 2.4 μg mL^−1^. After DOX and MCF-7/ADR cells were incubated together, the IC_50_ value was 83.5 μg mL^−1^. The results showed that MCF-7/ADR cells had high drug resistance, and the drug resistance index was 34.8. As shown in Fig. 2D, after incubating DOX@MOF-COD@CS nanoparticles with MCF-7/ADR cells, the IC_50_ value was 11.1 μg mL^−1^, the value was reduced by seven times compared to the value of free DOX. Therefore, it was proved that the nanosystem can reverse drug resistance. However, the other groups were incubated with MCF-7 cells and MCF-7/ADR cells, respectively, showed a lower inhibition rate compared with the final preparation (Additional file 1: Figs. S6 and S7). In addition, we investigated the toxicity of the nanosystem to normal human breast epithelial cells (HBL-100). It was proved that the carrier system did not damage normal cells and had good safety (Additional file 1: Fig. S5).

Cholesterol is rich in drug-resistant cell membranes and forms a hard lipid raft area. Cholera toxin subunit B (CTB) can specifically bind to the ganglioside GM1 in the lipid raft area. As shown in Fig. 2E, the strong green fluorescence can be observed in the control group, which was due to the lipid rafts formed by the rich cholesterol in drug-resistant cells. MOF-COD, DOX@MOF-COD@CS, M-β-CD treated cells, only faint green fluorescence was observed, indicating that cholesterol consumption can affect the formation of lipid rafts in the cell membrane and reduce the binding of AF488-CTB, the fluorescence intensity was weakened. The quantitative results of loss (Additional file 1: Fig. S8) are consistent with the laser confocal image.

As shown in Fig. 2F, this experiment used the cholesterol measurement kit to quantify cholesterol in drug-resistant cells. M-β-CD has the effect of consuming membrane cholesterol [50, 51]. As a positive control, it can be seen that the cholesterol content of the M-β-CD group was reduced to (47.6 ± 0.9)%, MOF-COD and DOX@MOF-COD@CS groups were reduced to (43.0 ± 1.0)% and (38.7 ± 1.2)%, respectively, the results of DOX@MOF-COD@CS group were significantly different from those of the control group (***P < 0.001). Use the fluorescent substance 1-pyridinedienoic acid as a probe to investigate the changes in fluidity (Fig. 2G). The fluorescent substance has main fluorescence emission peaks at 397 nm and 475 nm wavelengths (Additional file 1: Fig. S9). Among them, the wavelength of 397 nm corresponded to the monomer state of the fluorescent substance, and the wavelength of 475 nm corresponded to the state of excimer. When the fluorescent substance was in the state of excimer, it showed that the fluidity of cell membrane was better. After the nanoparticles interact with drug-resistant cells, the maximum fluorescence intensity ratio of the excimer to the monomer was 0.68 ± 0.02. Compared with β-CD, the ratio was 0.72 ± 0.02. There was no significant difference between the two groups, which proved that the nanoparticles had a similar fluidity regulation effect to M-β-CD and can enhance the fluidity of cell membranes.

In addition, it was investigated whether drug-resistant cells would transport the intracellular drug to the outside of the cells over time after the drug-resistant cells took up the drugs. As shown in Fig. 2H, 2 h after stopping ingestion, the drug accumulation of free DOX group in cells was 63.0%. Compared with the 2 h accumulation of the nano-system (95.6%), the efflux was 32.6%. While the intracellular accumulation of free DOX group continued to decrease at 4 h and 8 h. The drug efflux of free DOX group was 53.6% at 8 h, the value in DOX@MOF-COD@CS group was only 27.8%, which further showed that DOX@MOF-COD@CS nanoparticles can inhibit the drug from being excreted outside the cell. Further investigation of the expression of P-gp by western blot showed that the delivery system can significantly reduce the expression of efflux protein, thereby reducing drug efflux (Additional file 1: Fig. S10). On the one hand, the nanosystem catalyzed cholesterol, the lipid raft was destroyed, the rigidity was decreased, and the fluidity was increased, which was conducive to the continuous intake of the preparation. On the other hand, cholesterol was reduced, which was not suitable for P-gp to locate on the cell membrane, thereby increasing the accumulation of intracellular drugs and reversing drug resistance.

### Study on DOX@MOF-COD@CS nanoparticles inducing apoptosis of drug-resistant cells

**Fig. 3:**
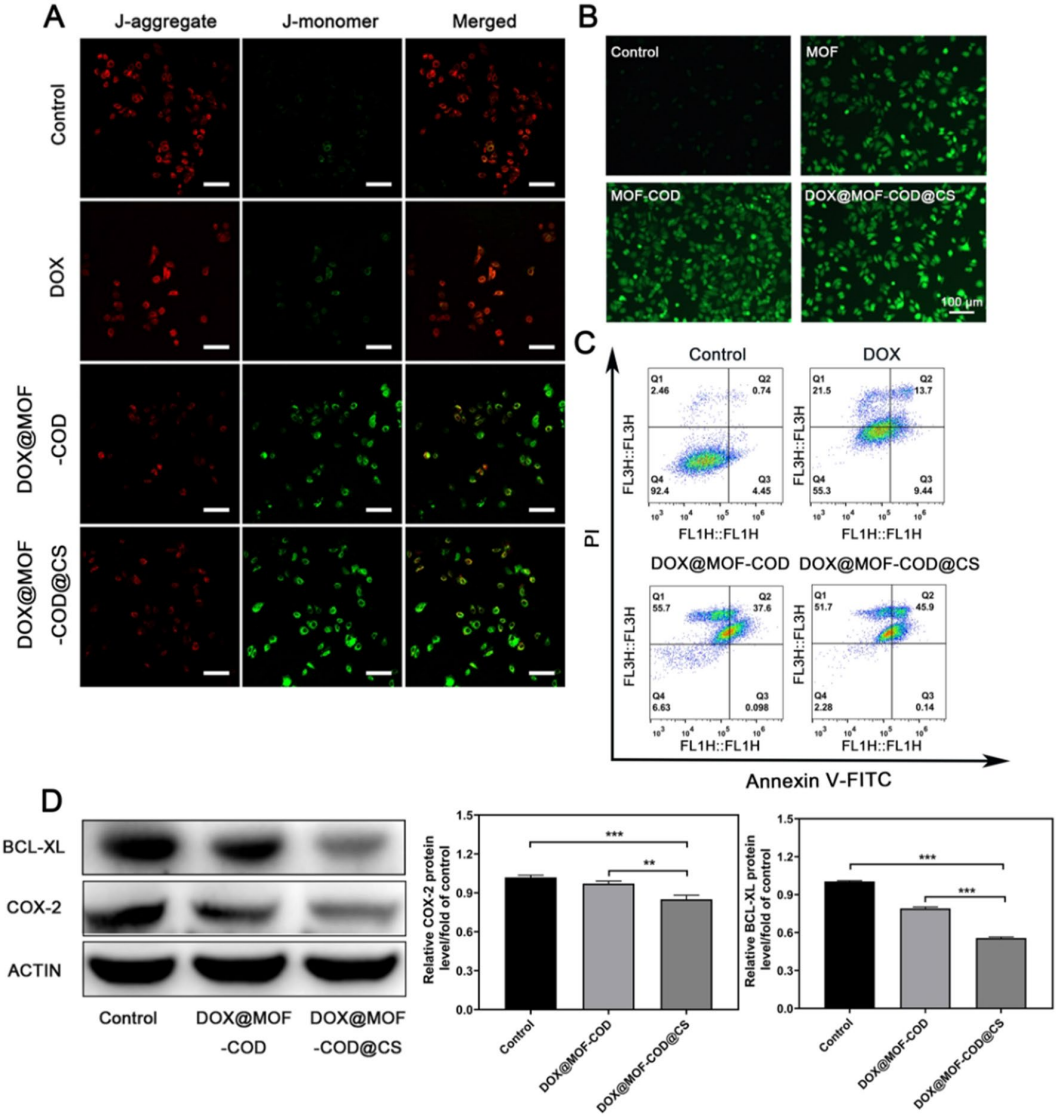
Research on apoptosis and protein. **A** Effects of different preparations on mitochondrial membrane potential of MCF-7/ADR cells. J-aggregates: Red fluorescent; J-1 monomer: Green fluorescence. **B** Results of DOX@MOF-COD@CS nanoparticles producing reactive oxygen species in MCF-7/ADR cell. DCFH was used as the probe. DCFH oxidation forms DCF: green fluorescence. **C** Annexin V-FITC/PI double staining method to determine the effects of different preparations on the cell apoptosis. **D** Western blot test and the related protein expression of COX-2 and BCL-XL. Data are presented as mean ± SD (*n* = 3), **P < 0.01, ***P < 0.001

In this experiment, the JC-1 fluorescent probe showed red and green fluorescence changes to investigate the different preparation effects on the cell apoptosis. The decreased mitochondrial membrane potential was a marker of apoptosis. When the mitochondrial membrane potential was higher, JC-1 aggregated in the matrix and formed a polymer with red fluorescence. When the mitochondrial membrane potential was low, JC-1 could not aggregate in the matrix of mitochondria and form monomer with green fluorescence. As shown in Fig. 3A, the green fluorescence of the control group was darker, indicated that the cells were not in apoptosis. Due to the poor fluidity of the drug-resistant cell membrane, DOX was difficult to be taken up by the cells and easily pumped out of cells by P-gp, which can only have a weak killing effect on the cells, and the green fluorescence was stronger than control group. As both DOX@MOF-COD and DOX@MOF-COD@CS groups consumed cholesterol and were easily taken up by MCF-7/ADR cells, the green fluorescence was significantly enhanced, the killing effects were improved remarkably. Also the reduced mitochondrial potential indicated that the cells were in apoptotic state. In order to verify that the DOX@MOF-COD@CS nanosystem can produce reactive oxygen species in MCF-7/ADR cells, as shown in Fig. 3B, the MOF group can catalyze intracellular H_2_O_2_ to generate hydroxyl radicals, showing green fluorescence. The MOF-COD and MOF-COD@CS groups can not only catalyze the breakdown of the intracellular H_2_O_2_, but also catalyze the H_2_O_2_ generated by cholesterol oxidase, the results showed stronger green fluorescence in DOX@MOF-COD@CS group than that of the MOF group. The DOX@MOD-COD@CS nanoparticles were incubated with the drug-resistant cells for 24 h, the cell apoptosis was obvious. The apoptotic rate of DOX@MOF-COD@CS group was 46.0%. The apoptotic rates of free DOX and DOX@MOF-COD were 23.1% and 37.7%, respectively. The changes of COX-2 and anti-apoptotic factor BCL-XL in the preparation group were further determined by western blot for 24 h since there was obvious apoptosis in the preparation group after 24 h treatment test. After the disulfide bond in the CS gel shell responded to GSH cleavage to release free CS, CS as an anti-inflammatory drug can directly inhibit the synthesis of pro-inflammatory factor cyclooxygenase COX-2, thereby reducing the level of anti-apoptotic protein BCL-XL and promoting cell apoptosis (Fig. 3D). These results were due to the consumption of cholesterol by MOF-COD nanoparticles and the increase of membrane fluidity.

### In vivo targeting and pharmacodynamic research

**Fig. 4:**
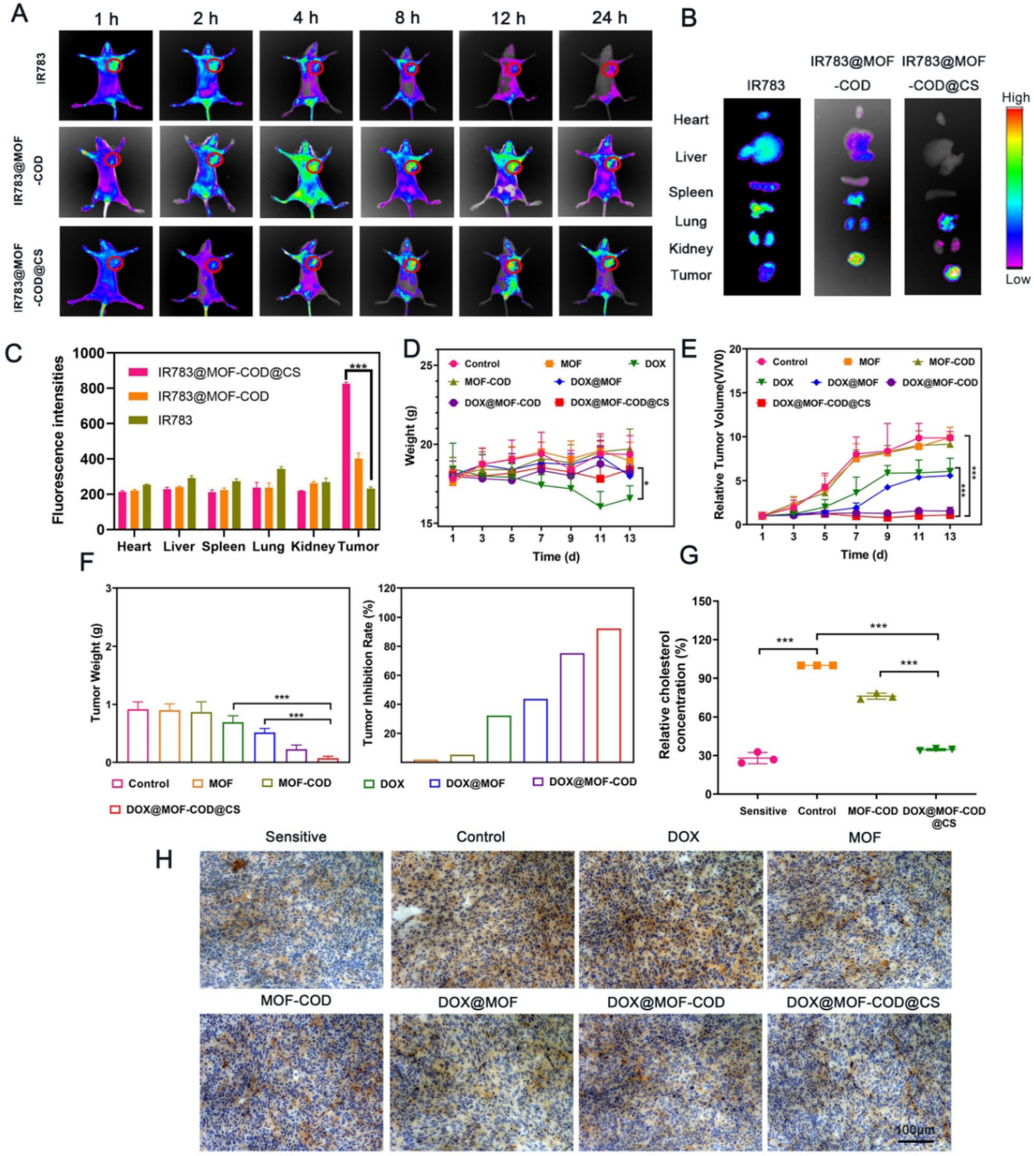
Distribution of IR783@MOF-COD@CS nanoparticles in tumor-bearing nude mice. **A** In vivo near-infrared fluorescence imaging of tumor-bearing nude mice labeled with IR783 and IR783@MOF-COD@CS through the tail vein at 1, 2, 4, 8, 12, 24, and 36 h, respectively (*n* = 3). **B** The results of fluorescence of the removed organs after 12 h of injection (*n* = 3). **C** The fluorescence intensity of IR783, IR783@MOF-COD and IR783@MOF-COD@CS in major organs. **D** The body weight change curves of different groups during treatment (*n* = 6). **E** Relative tumor volume change curve of each group (*n* = 6). **F** Tumor weight and inhibition rate of nude mice in each group. Data are presented as mean ± SD, *P < 0.05, **P < 0.01, ***P < 0.001. **G** Analysis of relative cholesterol content in tumor-bearing nude mice (sensitive group, MCF-7) and (drug-resistant group, MCF-7/ADR) (*n* = 3), **P < 0.01, ***P < 0.001. **H** After 14 days of treatment, the expression of P-gp in the parent tumor tissues (sensitive group, MCF-7) (drug-resistant group, MCF-7/ADR) was detected by immunohistochemistry

CS gel shell can specifically target tumor CD44 receptor and reduce damage to normal tissues. In order to study the targeting effect of the delivery system, the fluorescent dye IR783 was loaded into the carriers. The fluorescence intensity of free IR783 was low, and hardly any fluorescence was observed at 24 h, indicating that free IR783 was quickly eliminated (Fig. 4A). However, IR783@MOF-COD@CS group had strong fluorescence at the tumor site, and the fluorescence intensity of the tumor site can still be observed after 24 h. Free IR783 was rarely enriched in tumors, and mainly distributed in the organs such as liver and kidney, while IR783@MOF-COD@CS nanoparticles were less aggregation in other organs (Fig. 4B). The quantitative result (Fig. 4C) was consistent with the fluorescence signal. This result proved that the nano-delivery system had a good targeting and accumulation effect on tumor sites, and reduced damage to the normal tissues.

The tumor volume of nude mice in the saline group showed a rapid increase, followed by the MOF and MOF-COD groups (Fig. 4E). Because nude mice were drug-resistant model, the tumor volumes in DOX group and DOX@MOF group were large, the values were relatively small in DOX@MOF-COD and DOX@MOF-COD@CS groups. The body weights of the MOF and MOF-COD groups continued to increase, while the value of the free DOX group continued to decrease (Fig. 4D). This was due to the inability of free DOX to specifically target the tumor site and cause damage to normal tissues. The weight of the DOX@MOF-COD group and DOX@MOD-COD@CS group also showed an increasing trend. It is mainly because of the increased uptake of drugs, and improved anti-tumor effect.

The nude mice after the treatment were sacrificed, and the tumors were stripped and weighed and photographed (Additional file 1: Fig. S11). The tumor weight of the saline, MOF, and MOF-COD groups were about the same (Fig. 4F). The tumor weight of the DOX group was 0.69 ± 0.11 g, which may be due to the poor targeting of free DOX in vivo. Secondly, DOX was not easily taken up and excreted by drug-resistant cells, and the effect was not ideal. However, the tumor weight in the DOX@MOF-COD@CS group was significantly reduced, the value was (0.07 ± 0.03 g), and the tumor suppression rate was 92.2%. This is because the delivery system can specifically target the tumor site and consume cholesterol to increase the concentration of the drugs in the cells. Compared with free DOX group, it also has good safety in vivo and does not cause damage to organs (Additional file 1: Figs. S12, S13 and S14).

The amount of cholesterol in the body was quantitatively determined, and the results showed that the cholesterol content was reduced to 34.6% after the final preparation was applied (Fig. 4G). In order to investigate that the DOX@MOF-COD@CS nanosystem can reduce P-gp in vivo, this study investigated the expression of P-gp in each group through immunohistochemistry. P-gp located on the cell membrane was labeled brown using a primary antibody, and the browning on the membrane was reduced, indicating that P-gp was down-regulated (Fig. 4H). The control group showed a large amount of P-gp expression, and the DOX@MOF-COD@CS group showed a significant decrease in the expression level of P-gp.

### In vivo investigation of nanoparticle-induced cell apoptosis

**Fig. 5:**
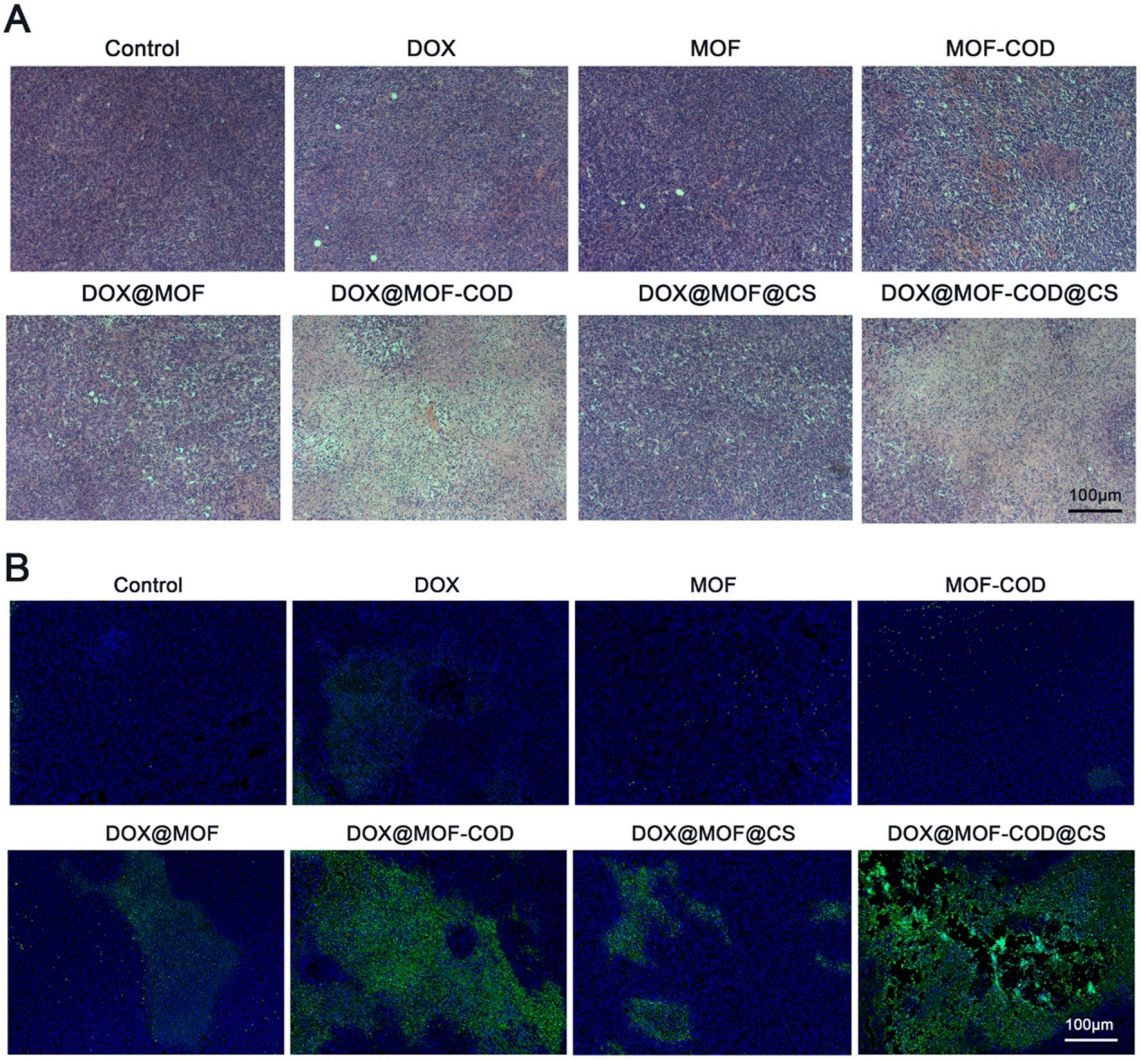
In vivo tumor tissue suppression effect evaluation. **A** H&E staining images of tumor tissues in each group. **B** TUNEL staining to detect tumor tissue apoptosis

Observe the tumor slices of each group to evaluate the anti-tumor effect of the DOX@MOF-COD@CS nanosystem. The H&E staining of the tumor tissues in each group showed that the nuclei of saline group were dense and intact. In the DOX@MOF-COD@CS group, the significant nucleus shrinkage, the reduced cell arrangement density, and severe necrosis were observed. The above results all indicated that the delivery system has a significant tumor suppressing effect in vivo (Fig. 5A). There was no significant apoptosis in DOX group, which was similar to the control group, indicating that the free DOX group was difficult to be taken up by the drug-resistant cells, while the DOX@MOF-COD and DOX@MOF-COD@CS groups can increase the uptake of drugs, and show strong green fluorescence, which indicated the delivery system can promote the cell apoptosis (Fig. 5B).

## Conclusion

In short, we cleverly constructed a nano-delivery system based on enzymatic reaction (DOX@MOF-COD@CS). This system can specifically target tumor CD44 receptors and respond to GSH cleavage. On the one hand, CS can inhibit the synthesis of the pro-inflammatory enzyme COX-2, leading to the down-regulation of the anti-apoptotic protein BCL-XL, thereby increasing the sensitivity of tumor cells to chemotherapeutics. On the other hand, the nanoenzymes and natural enzymes initiate cascade catalysis. The cholesterol metabolite H_2_O_2_ is catalyzed by cholesterol oxidase, which can be further catalyzed by nanoenzyme MOF to produce •OH. Cholesterol, which is beneficial to the resistance of drugs, can be transformed into •OH, which can kill cancer cells, promote cell apoptosis and reverse drug resistance to the greatest extent. Therefore, taking drug-resistant cell membranes as a "breakthrough", combined with the anti-apoptotic pathway in the drug resistance mechanism, this research will provide a new idea for reversing multidrug resistance in tumors.

## Supplementary Information

**Additional file 1. **Supplementary enzyme activity of the preparation, in *vitro* and in vivo experimental data.
