# Supplementary material for: Nanozyme-natural enzymes cascade catalyze cholesterol consumption and reverse cancer multidrug resistance
Source: J Nanobiotechnology. 2022 May 2;20:209. doi: 10.1186/s12951-022-01406-9 (PMC9063293; doi:10.1186/s12951-022-01406-9)
Supplement: Supplementary file 1 — Additional file 1. Supplementary enzyme activity of the preparation, in vitro and in vivo experimental data. [file 12951_2022_1406_MOESM1_ESM.docx]

Additional file 1

**Nanozyme-natural enzymes cascade catalyze cholesterol consumption and reverse cancer multidrug resistance**

*Bin Du ^a,b^, Mei Zheng ^a^, Huizhen Ma ^a^, Jingshu Huang ^a^, Qingqing Jiao ^a^, Yimeng Bai ^a^, Mengmeng Zhao ^a^, Jie Zhou ^a,b,*^*

^a^ *School of Pharmaceutical Sciences, Zhengzhou University, 100 Science Road, Zhengzhou 450001, P. R. China*

^b^ *Key Laboratory of Targeting Therapy and Diagnosis for Critical Diseases, Henan Province, 100 Science Road, Zhengzhou 450001, P. R. China*

^*^Corresponding author.

E-mail address: [zhj_paper@sina.com](mailto:zhj_paper@sina.com)


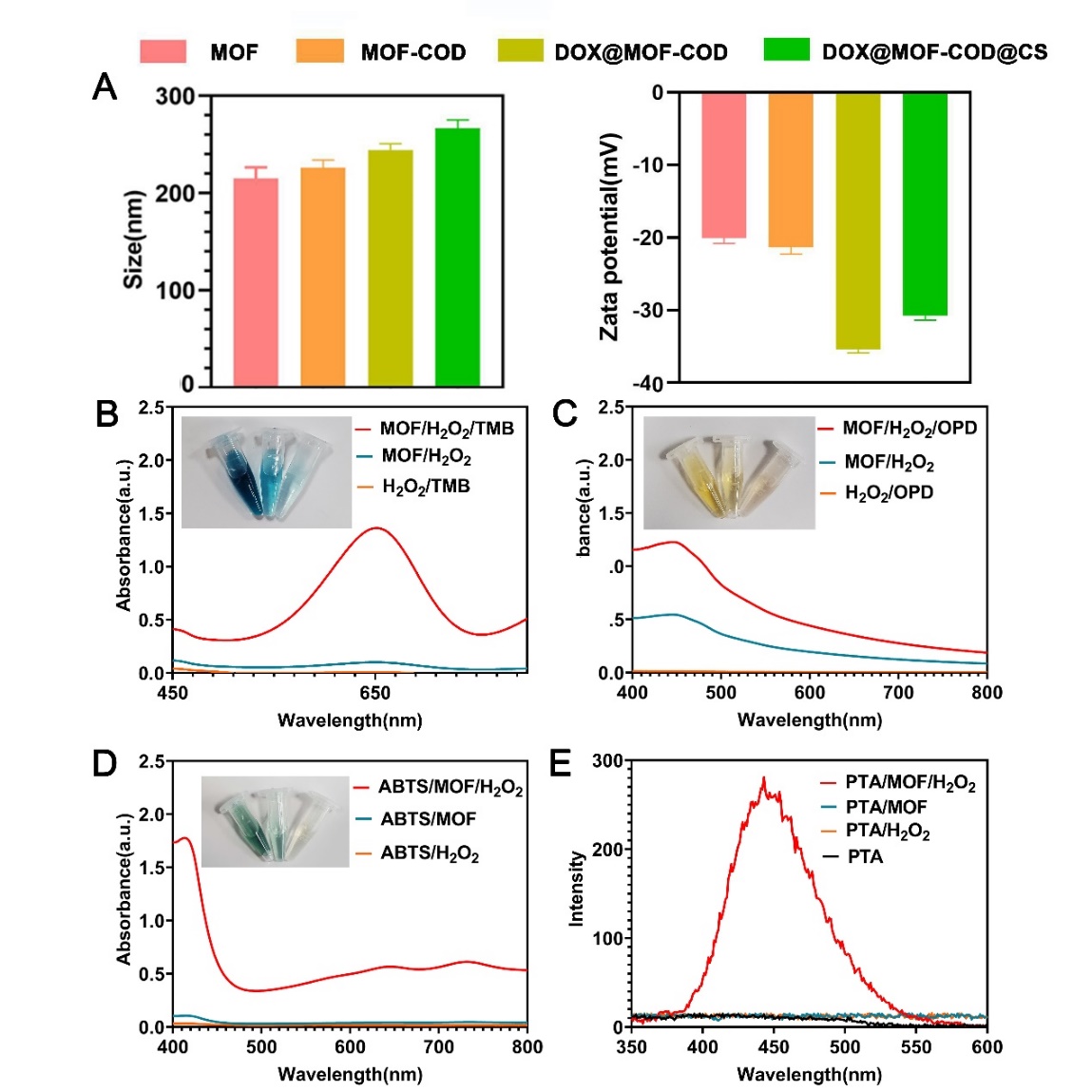


**Fig. S1** Characterizations of the different nanoparticles. (A) Particle sizes and zeta potentials of MOF, MOF-COD, DOX@MOF-COD, DOX@MOF-COD@CS nanoparticles. (B-E) Peroxidase-like properties of MOF. ([MOF] = 40 μg·mL^-1^, [H_2_O_2_] = 200 μM, pH = 4, [TMB] = 1mM, [ABTS] = 1mM, [OPD] = 1mM, [PTA] = 1 mM, temperature: 37℃)


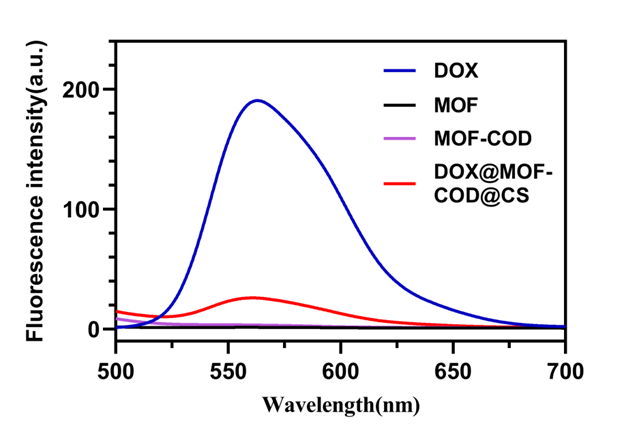


**Fig. S2** Fluorescence emission spectra of DOX, MOF, MOF-COD and DOX@MOF-COD@CS nanoparticles.


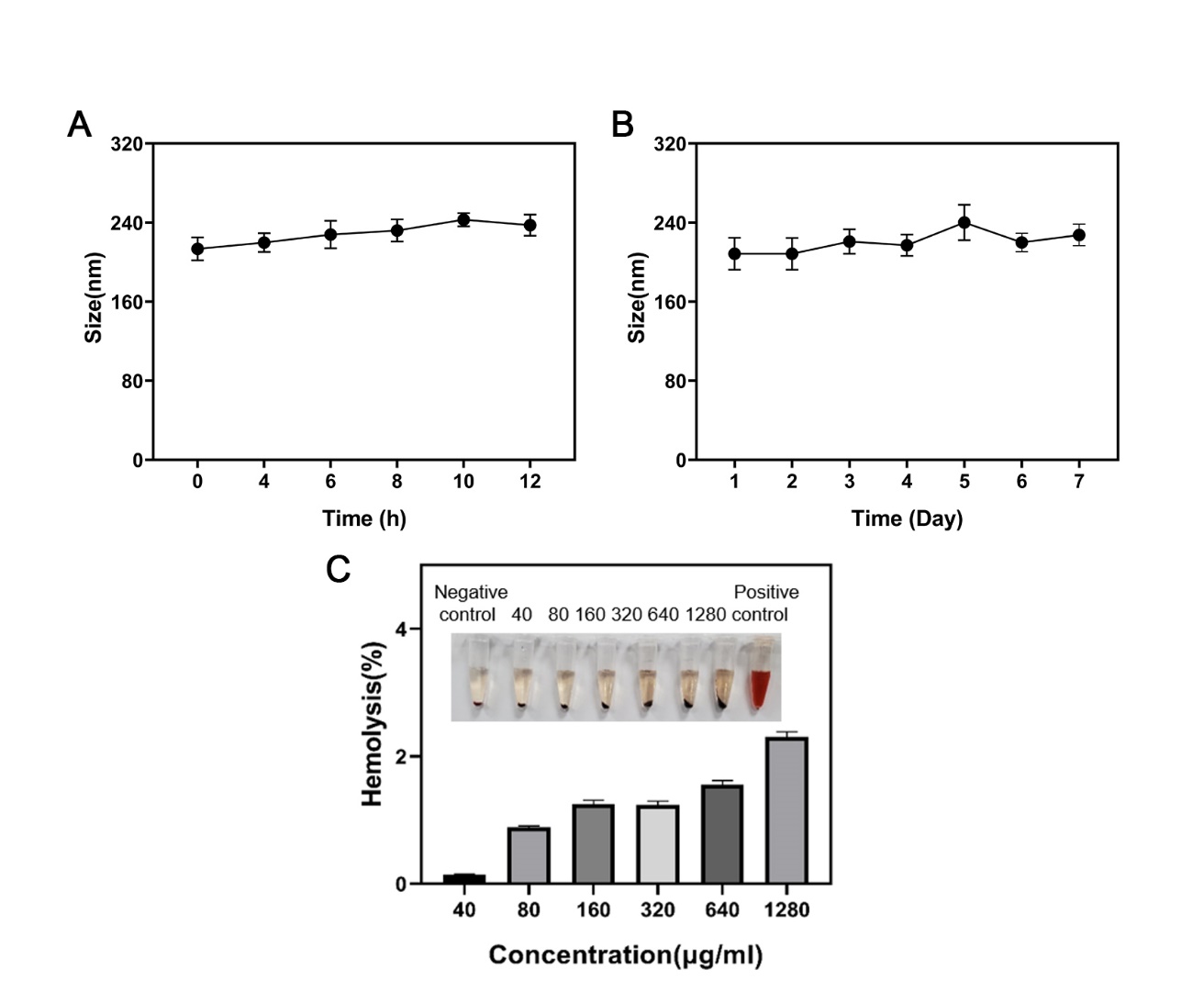


**Fig. S3** Researches on the stability and security of DOX@MOF-COD@CS nanoparticles. (A) Changes in the sizes of DOX@MOF-COD@CS nanoparticles incubated in serum for 24 h. (B) Changes in the sizes of DOX@MOF-COD@CS nanoparticles incubated in PBS for 7 days. (C) Hemolysis test of DOX@MOF-COD@CS nanoparticles.


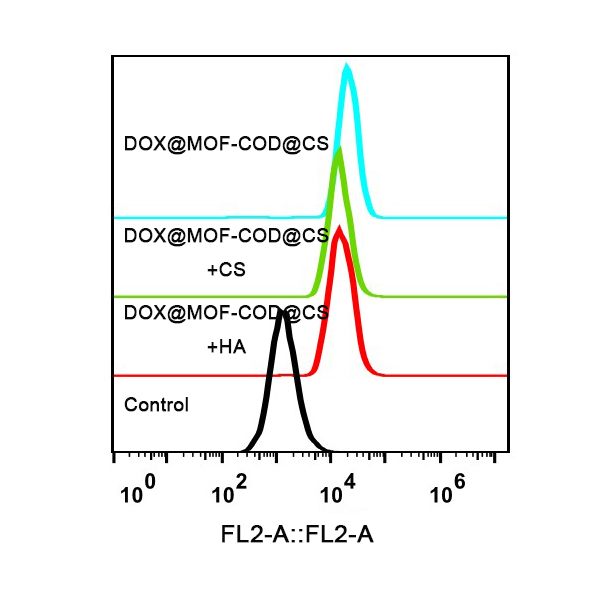


**Fig. S4** Flow cytometry analysis of the targeting effect of different nanoparticles on CD44.


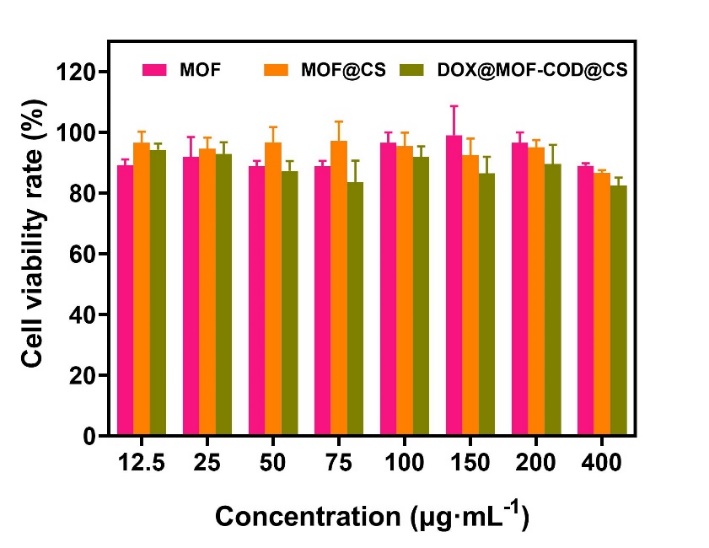


**Fig. S5** Cell viability rate of HBL-100 incubated with MOF, MOF@CS, and DOX@MOF-COD@CS nanosystem for 48 h, there was no significant difference between the groups (*n* = 6).


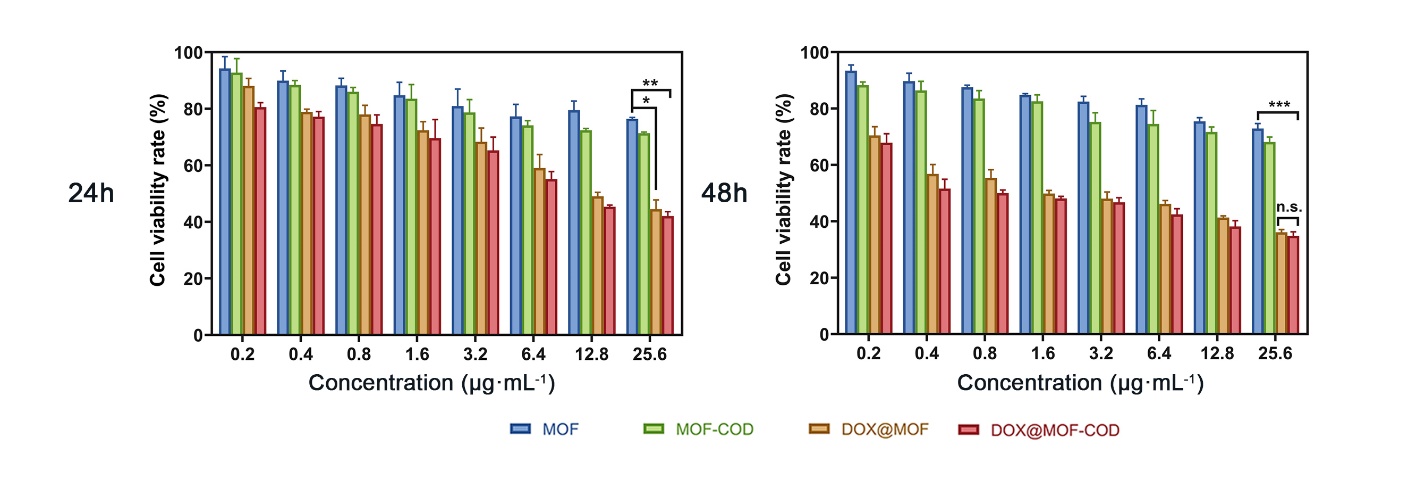


**Fig. S6** Cell viability rates of MCF-7 cells incubated with MOF, MOF-COD, DOX@MOF, DOX@MOF-COD for 24 and 48 h (*n* = 6), **P* <0.05, ***P* <0.01, ****P* <0.001.


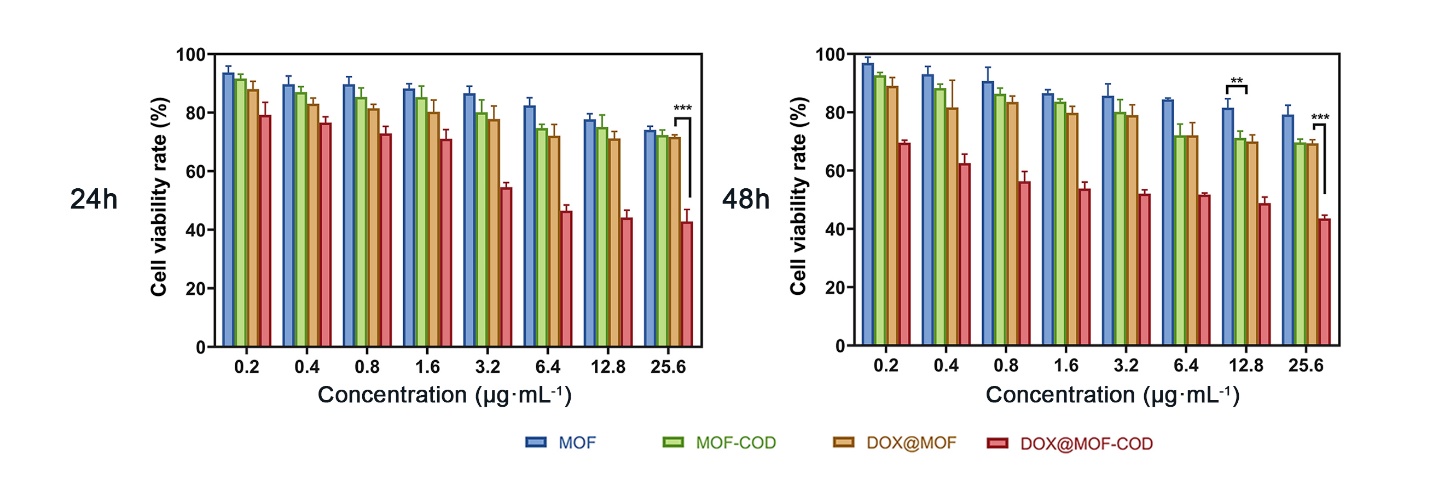


**Fig. S7** Cell viability of MCF-7/ADR cells incubated with MOF, MOF-COD, DOX@MOF, DOX@MOF-COD nanoparticles for 24 and 48 h (*n* = 6), data are expressed as means ± SD, ***P* <0.01, ****P* <0.001.


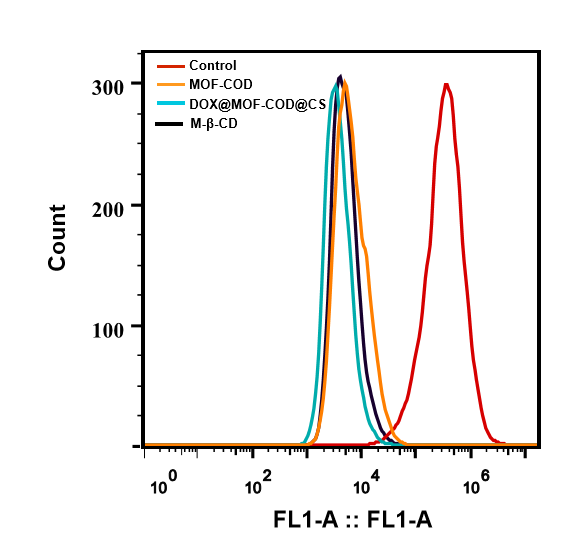


**Fig. S8** Fluorescence intensity of lipid rafts was analyzed by flow cytometry.


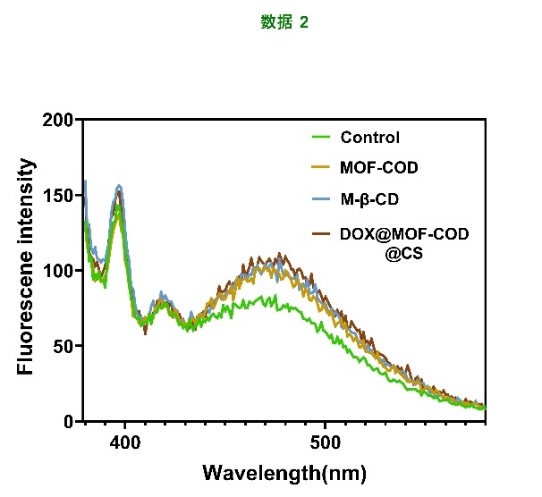


**Fig. S9** Fluorescence emission spectra of 1-pyrenedodecanoic acid.

**
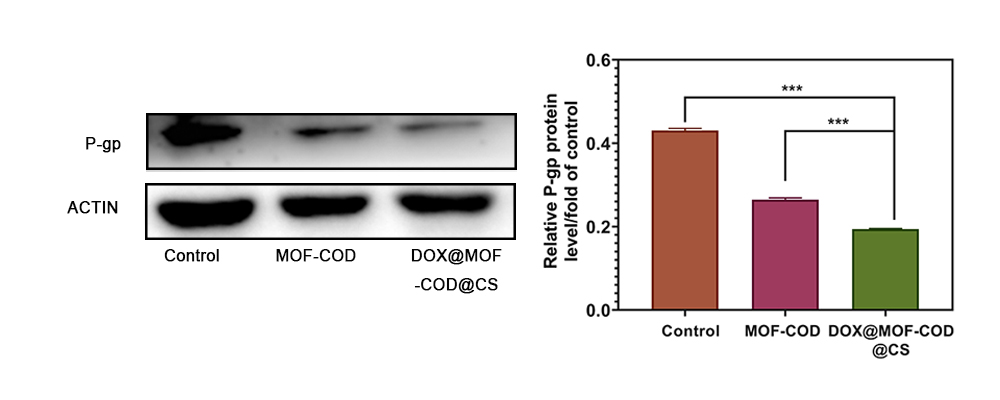
**

**Fig. S10** Western blot to study the content of P-gp and gray-scale analysis of P-gp protein (*n* = 3), ****P* <0.001.


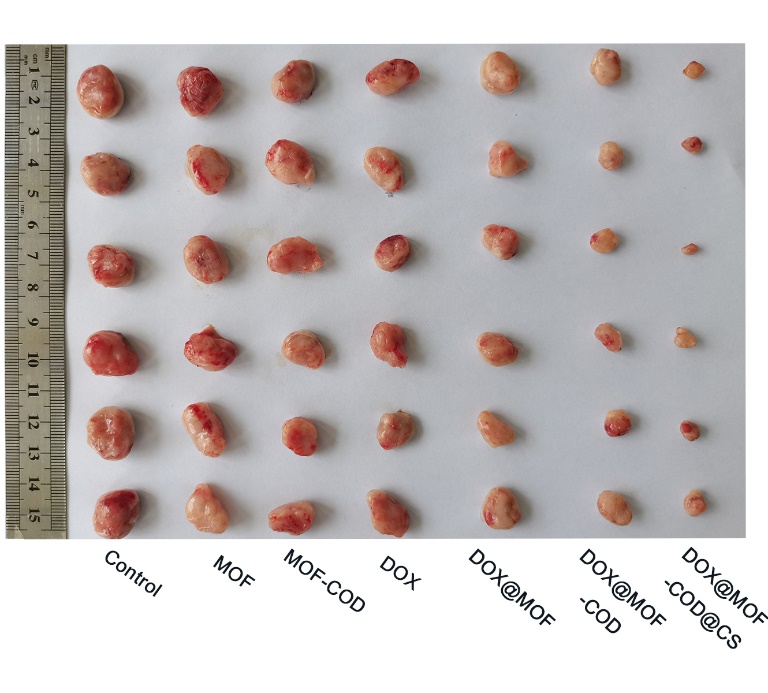


**Fig. S11** Photographic records of nude mice tumors in each group.


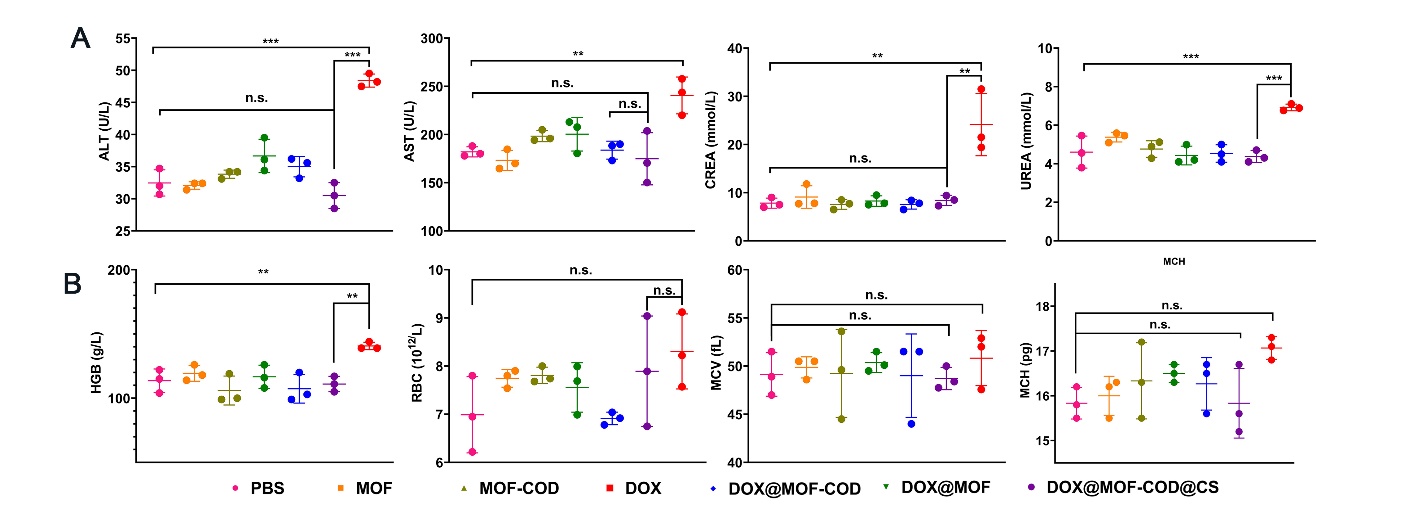


**Fig. S12** *In vivo* safety evaluation. (A) Detection of liver and kidney function in each group. Alanine aminotransferase (ALT), aspartate aminotransferase (AST), creatinine (CREA). (B) Blood routine test, hemoglobin (HGB), red blood cell (RBC), mean corpuscular volume (MCV), mean corpuscular hemoglobin (MCH) (n= 3), **P <0.01, ***P <0.001.


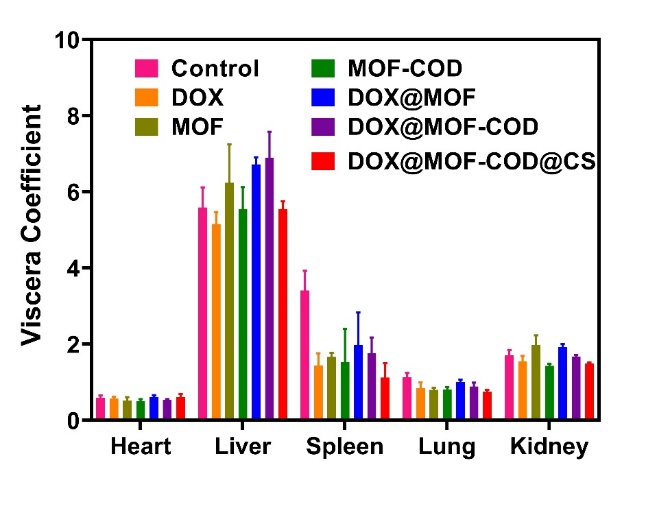


**Fig. S13** Investigation of organ index in each group (*n*= 6).


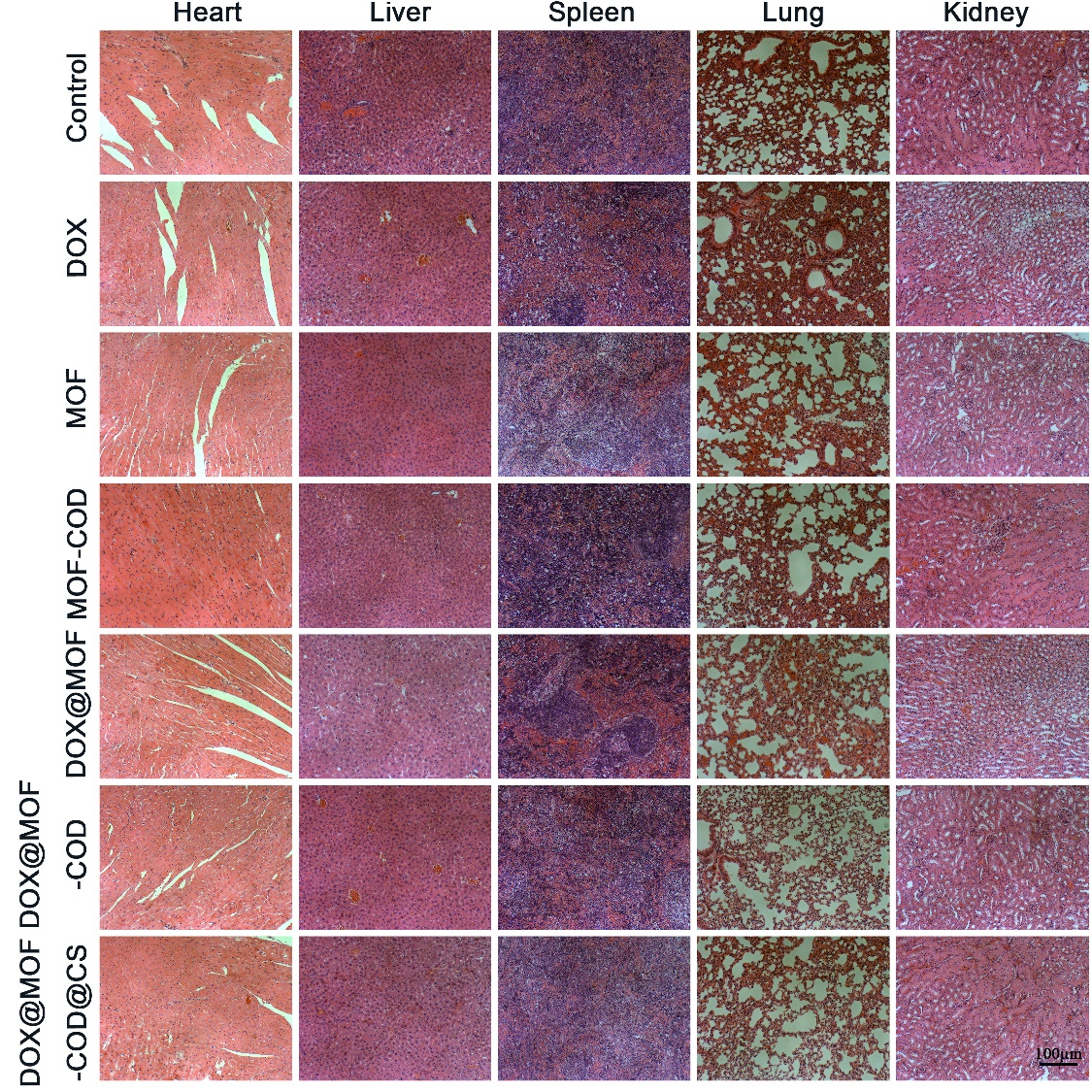


**Fig. S14** H&E staining study on major organs (Heart, Liver, Spleen, Lung, Kidney).
